# Association between *APOE* e4 and white matter hyperintensity volume, but not total brain volume or white matter integrity

**DOI:** 10.1007/s11682-019-00069-9

**Published:** 2019-03-22

**Authors:** Donald M. Lyall, Simon R. Cox, Laura M. Lyall, Carlos Celis-Morales, Breda Cullen, Daniel F. Mackay, Joey Ward, Rona J. Strawbridge, Andrew M. McIntosh, Naveed Sattar, Daniel J. Smith, Jonathan Cavanagh, Ian J. Deary, Jill P. Pell

**Affiliations:** 1 Institute of Health & Wellbeing, University of Glasgow, 1 Lilybank Gardens, G12 8RZ, Glasgow, Scotland, UK; 2 Centre for Cognitive Ageing and Cognitive Epidemiology, University of Edinburgh, Scotland, UK; 3 Institute of Cardiovascular and Medical Sciences, University of Glasgow, Scotland, UK; 4 Department of Medicine Solna, Karolinska Institute, Stockholm, Sweden

**Keywords:** Aging, APOE, Epidemiology, Genetic association studies, MRI

## Abstract

Apolipoprotein (*APOE*) e4 genotype is an accepted risk factor for accelerated cognitive aging and dementia, though its neurostructural substrates are unclear. The deleterious effects of this genotype on brain structure may increase in magnitude into older age. This study aimed to investigate in UK Biobank the association between *APOE* e4 allele presence vs. absence and brain imaging variables that have been associated with worse cognitive abilities; and whether this association varies by cross-sectional age. We used brain magnetic resonance imaging (MRI) and genetic data from a general-population cohort: the UK Biobank (*N* = 8395 after exclusions). We adjusted for the covariates of age in years, sex, Townsend social deprivation scores, smoking history and cardiometabolic diseases. There was a statistically significant association between *APOE* e4 genotype and increased (i.e. worse) white matter (WM) hyperintensity volumes (standardised beta = 0.088, 95% confidence intervals = 0.036 to 0.139, *P* = 0.001), a marker of poorer cerebrovascular health. There were no associations with left or right hippocampal, total grey matter (GM) or WM volumes, or WM tract integrity indexed by fractional anisotropy (FA) and mean diffusivity (MD). There were no statistically significant interactions with age. Future research in UK Biobank utilising intermediate phenotypes and longitudinal imaging hold significant promise for this area, particularly pertaining to *APOE* e4’s potential link with cerebrovascular contributions to cognitive aging.

**Electronic supplementary material:**

The online version of this article (10.1007/s11682-019-00069-9) contains supplementary material, which is available to authorized users.

## Introduction

Variation at the *APOE* genetic locus is an established risk factor for Alzheimer’s disease (AD) (Lutz et al. 2010), and cognitive decline in domains of memory, information processing speed and overall cognitive function (‘*g*’) (Wisdom et al. 2011). The e4 allele, which typically has a frequency of around 15% in Caucasian/European populations (Eisenberg et al. 2010), is known as the ‘risk’ allele, vs. the neutral e3 allele (typical frequency 78%) and possibly protective e2 allele (frequency 6%). The *APOE* locus’s main function relates to lipid/cholesterol metabolism, which is pleiotropic for several biological functions including neuronal migration, axon guidance, and the clearance of amyloid beta plaques – which characterise AD - in the brain (Holtzman et al. 2012).

There is evidence that the effects of *APOE* e4 variation on brain functioning increase across the lifespan, i.e. differences between e4 carriers vs. non-carriers in terms of cognitive ability become more pronounced with older age regardless of outright dementia (Schiepers et al. 2011). Davies et al. (2015) reported data from the CHARGE consortium where in 53,949 European participants from 31 different cohorts (aged >45 years and non-demented), the strength of association between rs10119 (in the *APOE* genetic region) and a general factor of fluid cognitive function, increased linearly with the mean age of each cohort (Pearson’s *r* = −0.42, *P* = 0.022). Similarly, a report that included data from the Generation Scotland cohort (Marioni et al. 2015) (*n* = 18,337) reported significant negative associations between *APOE* e4 allele presence and tests scores on Logical memory (standardized beta = −0.095, *P* = 0.003), and Digit symbol coding (standardized beta = −0.087, *P* = 0.004); however this was only significant in participants aged 60 years or over. We recently investigated potential *APOE* e4 genotype-by-age interaction on cognitive function in UK Biobank (n = ~111 k) (Lyall et al. 2016b); there were no statistically significant interactions; however there are concerns over the potential imprecision and reliability in the novel UK Biobank baseline cognitive tests (Lyall et al. 2016a), which means that result may be an underestimate of the potential true effect. Overall, recent research warrants further study into the potential structural brain substrates of the *APOE* e4 effect into older age.

It is important to understand the neurobiological underpinnings of the potential age-related deleterious effects of *APOE* e4 on brain function. The integrity of connective WM tracts in the brain is associated with overall cognitive function (Penke et al. 2012). The hippocampus is a known brain substrate of memory loss, one of the key symptoms of AD, and is the first site to show evidence of amyloid beta plaques (Reilly et al. 2003). Total brain volumes, including total GM and WM volumes are significantly associated with changes in cognitive function (Royle et al. 2013). Brain WM hyperintensities are a major substrate of age-related cognitive decline and cerebrovascular disease (Debette and Markus 2010), and common in AD (Paternoster et al. 2009).

Investigating associations between *APOE* genotype and MRI markers is a topic of intense research, but studies involving structural neuroimaging parameters have not produced consistent results (Scarmeas and Stern 2006). Whereas e4 carriers appear to be at increased risk of hippocampal atrophy (Manning et al. 2014), worse WM microstructural integrity (Slattery et al. 2017) and selective GM atrophy (Pievani et al. 2009) in the context of pathological aging (such as AD and mild cognitive impairment), there is less clarity with respect to MRI markers in non-pathological aging. For example, null associations are reported for *APOE* genotype and cross-sectional hippocampal volume (Ferencz et al. 2013; Jack et al. 1998; Killiany et al. 2002; Lyall et al. 2013; Manning et al. 2014; Schuff et al. 2009), brain atrophy (Cherbuin et al. 2008), GM volume (Cherbuin et al. 2008) and some WM measures (Lyall et al. 2014, 2015), though *APOE* e4 carriers exhibited greater WM hyperintensity growth over a 3-year period in older age when compared to non-e4 carriers (Cox et al. 2017). A meta-analysis (*n* = 8917) reported that e4 genotype is associated with greater burden of MRI markers associated with cerebrovascular disease (cerebral microbleeds (Schilling et al. 2013)). However, many prior studies possess low statistical power with which to detect subtle effects specifically in mid- and later-life, and thus it is unclear whether *APOE* status is unrelated to non-pathological brain aging, or whether subtler differences exist which prior studies have been unable to reliably quantify. It is possible on the other hand that *APOE* e4 has a stable association with poorer brain health which is not more pronounced in older age (Heise et al. 2011).

The UK Biobank cohort is a large prospective cohort of whom at the time of writing around 13,000 have genetic and MRI data (prior to exclusions). This report aims to test for an effect of *APOE* e4 genotype carrier status on specific brain imaging phenotypes in UK Biobank, and whether that association interacts with age. The large sample N improves the potential reliability of association estimates herein, compared with smaller reports as well as statistical power to detect small effects. We examined several specific brain imaging phenotypes based on a-priori hypotheses described above: left and right hippocampal volumes, total GM and WM volumes, WM hyperintensity volumes, and brain white matter tract integrity metrics.

It is hypothesized that for each brain phenotype, the association between *APOE* e4 genotype will be in the deleterious direction (i.e. lower scores for all except WM hyperintensity volumes and tract mean diffusivity, for which higher scores are worse), and interact with age where the association becomes larger in older participants (on average). We assessed the phenotypes of: two latent measures of WM tract integrity derived from tract-specific FA (‘gFA’) and MD (‘gMD’); total GM; total WM; total WM hyperintensity volume and hippocampal volumes (all volumes in millimetres^3^). This will enable us to quantify, with considerable statistical power whether the effect of *APOE* e4 genotype on brain phenotypes of relevance to cognitive decline and AD is stronger in older age.

## Methods

### Study design and participants

The UK Biobank cohort is a large prospective cohort of 502,628 participants with phenotypic information. All participants attended one of 22 assessment centres from 2006 to 2010 where they completed a series of physical, sociodemographic, and medical assessments (Sudlow et al. 2015). In 2014, MRI scanning of baseline participants began, and this is ongoing until around 100,000 participants have been scanned. As of November 2017, *n* = 12,931 have MRI data derived by UK Biobank.

### Genetic data

UK Biobank genotyping was conducted by Affymetrix using a bespoke BiLEVE Axiom array for ∼50,000 participants and the remaining ∼450,000 on the Affymetrix UK Biobank Axiom array. All genetic data were quality controlled by UK Biobank (Bycroft et al. 2017). The *APOE* e genotype is directly genotyped. Further information on the genotyping process is available (http://www.ukbiobank.ac.uk/scientists-3/genetic-data), including detailed technical documentation (https://biobank.ctsu.ox.ac.uk/crystal/docs/genotyping_sample_workflow.pdf). The two *APOE* e SNPs – rs7412 and rs429358 – were both in Hardy Weinberg equilibrium (*P* > 0.05) assessed with PLINK V1.90 (Purcell et al. 2007).

### MRI data

An average of four years after initial recruitment, a subset of UK Biobank participants also underwent head MRI on the same scanner at a single site (Cox et al. 2016). The release of brain MRI data as of August 2017 is the subject of the current study. All brain imaging data used here was processed and quality checked by UK Biobank (Alfaro-Almagro et al. 2017; Miller et al. 2016) and available in the form of Imaging Derived Phenotypes (IDPs). Details on the UK Biobank imaging acquisition and processing including WM/GM and hippocampal segmentation, and on the WM diffusion processing, are freely available from three sources: the UK Biobank protocol: http://biobank.ctsu.ox.ac.uk/crystal/refer.cgi?id=2367 and documentation: http://biobank.ctsu.ox.ac.uk/crystal/refer.cgi?id=1977 and has been described elsewhere (Cox et al. 2016; Miller et al. 2016). The total WM and GM variables were segmented automatically using FAST (Zhang et al. 2001) and were normalised for skull size based on the T1 MR scan (see open-access MRI protocol, pp.11; https://biobank.ctsu.ox.ac.uk/crystal/docs/brain_mri.pdf). Total WM hyperintensity volumes were calculated based on T1 and T2 FLAIR, derived by UK Biobank using the Brain Intensity Abnormality Classification Algorithm (BIANCA) (Griffanti et al. 2016) with the procedure detailed by Miller et al. (2016). WM hyperintensity volumes were log-transformed here due to a positively skewed distribution.

FA and MD are commonly-derived WM tract integrity variables which describe the directional coherence and magnitude of water molecule diffusion, respectively. Water molecules tend to diffuse with greater directional coherence and lower magnitude when constrained by tightly-packed fibres (such as well-myelinated axons) as well as by cell membranes, microtubules and other structures; lower FA and higher MD are generally considered to reflect poorer/less ‘healthy’ white matter (Jones et al. 2013). We constructed general factors of FA (gFA) and MD (gMD) using principal components analysis based on 22 tracts (Cox et al. 2016). These general measures reflect the high degree of shared microstructural properties across major white matter tracts in the brain; as found in this cohort, and various other groups (Alloza et al. 2016; Cox et al. 2016; Penke et al. 2012; Telford et al. 2017). Inspection of eigenvalues showed clear single unrotated factor solutions for gFA (eigenvalue = 12.83, r^2^ = 58%) and gMD (eigenvalue = 13.76, r^2^ = 63%). All brain MRI metrics were transformed into Z-scores based on the final analysis sample to ease interpretation.

### Covariates

UK Biobank derived a Townsend deprivation score for all participants immediately prior to baseline; calculated from data on car ownership, household overcrowding, owner-occupation and unemployment aggregated for postcodes of residence (Townsend 1998). Higher Townsend scores equate to higher levels of area-based socioeconomic deprivation. Participants were asked during the baseline and MRI assessments about any previous or current cardiometabolic conditions that had been diagnosed by their doctor. Specifically, participants were asked whether their doctor had diagnosed each of myocardial infarction, angina, hypertension, diabetes or stroke (individually). We defined CHD as either myocardial infarction (MI) or angina. Smoking was classified as never, previous, or current smoker based on self-report; we simplified this into a binary ever (previous plus current) vs. never variable. We excluded participants that stated only ‘prefer not to answer’ for disease and smoking variables: less than 1% of the sample.

### Exclusions

There were 12,662 participants with *APOE* e genotype and brain MRI data. We excluded participants with non-white British ancestry, self-report vs. genetic sex mismatch, putative sex chromosomal aneuploidy, excess heterozygosity, and missingness rate > 0.1. This left *n* = 11,065. We removed participants who reported a neurological condition at baseline or scan visit (~5%; Supplementary Table 1); the inclusion of which could drive type-1 errors due to skewed results. We accounted for relatedness between participants by selecting one random participant for analysis from sets where two or more individuals were 1st cousins or closer. This left 8395 participants for whom genotype frequencies of *APOE* e were e2/e2 *n* = 48 (1%), e2/e3 *n* = 1032 (12%), e2/e4 = 208 (2%), e3/e3 = 4960 (59%), e3/e4 *n* = 1958 (23%) and e4/e4 *n* = 189 (2%). As a check, participants were split into age groups (under 50; 50 to 59; 60 and over): a chi square test showed no significant difference in e4 frequency (*p* = 0.148). These statistics are shown in Supplementary Table 2.

### Analysis

We used an e4+ dominant model of e3/e4 and e4/e4 collated vs. e2/e2, e2/e3 and e3/e3 collated; e2/e4 is usually removed because it has potentially risk and protective alleles (Wisdom et al. 2011). We elected for an e4 dominant (i.e. present vs. absent) rather than dose model (i.e. 0/1/2) because there were relatively few e4 homozygotes. We ran two linear regression models to examine associations between e4 genotype and each of the brain imaging parameters: gFA scores, gMD scores, left/right hippocampal volume, total GM, total WM and log WM hyperintensity volumes in mm^3^, each transformed to Z-scores. For the ‘partially adjusted model’ we adjusted for age, genetic array, 8 principal components (PCs) and sex. (The PCs were added conservatively to account for possible population stratification, although this made no difference to the results in terms of *APOE* e4). For the ‘fully adjusted model’ we then additionally adjusted for Townsend deprivation scores, ever vs. never smoking cigarettes, and self-reported doctor diagnosis of diabetes, hypertension and CHD. For interaction models we added the requisite e4*age interaction term. Finally, we re-ran analyses with added age^2^ and an e4*age^2^ interaction term to capture potentially curvilinear relationships.

We determined an alpha level of *P* < 0.05 as nominal significance, and corrected for multiple comparisons with the false discovery rate (FDR) using a specialized program (Pike 2011). We calculated statistical power using G*power 3 (Faul et al. 2007). We conducted two additional post-hoc sensitivity analyses: firstly, we adjusted for X/Y/Z-coordinates of brain position during scanning, because it was available in only *n* = 6647 participants. Secondly, we adjusted the final results for baseline assessment centre (one of 22 across the United Kingdom), to reduce the possibility of any systematic procedural differences e.g. handling of blood samples (although note that the MRI scanning data we report on here was single-site).

A significant effect of e4 allele presence vs. absence would indicate mean level differences in the brain parameter of interest as a function of *APOE* e genotype (vs. absence) cross-sectionally. A significant interaction with age (or age^2^) would be consistent with a hypothesis of greater (i.e. accelerating) decline with increasing age, according to *APOE* e4 allele presence vs. absence.

## Results

Descriptive statistics are shown in Table 1, including chi square (for ordinal/categorical data) and ANOVA (for continuous data) tests of differences between *APOE* e4 presence vs. absence groups. There were 8395 participants (mean age = 61.55, SD = 7.03, range = 46–73), of whom 3953 were male (46.1%). G*Power 3 showed we had 99% power to find a standardised effect size of Cohen’s *d* = 0.1 (where 0.2 is considered small) for significant associations or interactions, based on our e4 present vs. absent sample sizes.

**Table 1:** Descriptive statistics

	e4 absent (*N* = 6040; 74%)	e4 present (*N* = 2147; 26%)	Effect size	P value
*Demographic/lifestyle variables*				
Age in years, mean (SD)	61.66 (7.03)	61.19 (7.04)	0.07	0.009
Gender, male N (%)	2916 (48.28)	941 (43.83)	0.04	<0.001
Townsend score, mean (SD)	−2.035 (2.58)	−1.98 (2.58)	0.02	0.367
Smoking history, ever N (%)	2249 (40.32)	848 (39.55)	0.07	0.536
*Brain MRI values*				
Left hippocampal volume (mm^3^)	3821.69 (468.75)	3814.99 (446.79)	0.01	0.613
Right hippocampal volume (mm^3^)	3933.61 (480.72)	3924.82 (478.75)	0.02	0.521
Normalised total grey matter (mm^3^)	796,681.00 (47,417.56)	800,931.70 (46,699.26)	0.09	0.002
Normalised total white matter (mm^3^)	711,833.90 (40,033.25)	713,252.30 (41,790.69)	0.04	0.220
White matter hyperintensity vol. (mm^3^) [before log-transform]	3517.85 (4480.04)	3710.22 (4657.41)	0.04	0.157
*Disease prevalence rates*				
Coronary heart disease N, %	194 (3.22)	59 (2.75)	0.01	0.287
Hypertension N, %	1387 (22.99)	492 (22.95)	<0.01	0.971
Diabetes N, %	298 (4.97)	85 (4.00)	0.02	0.067

Effect size = Cohen’s *d* for continuous data; Cramer’s *V* for frequency data

There were no associations between *APOE* e4 and gFA, gMD (Table 2), left/right hippocampal volumes, total GM or total WM both normalised for skull size: indicating no mean-level difference in these brain parameters across the sample. There was a significant association between *APOE* e4 possession and greater WM hyperintensity volume (fully-adjusted standardised beta = 0.088, 95% CI = 0.036 to 0.139, *P* = 0.001). There were no significant e4*cross-sectional age interactions at *P* < 0.05. Results were unchanged when we added an age^2^ term (i.e. non-linear), adjusted for X/Y/Z-coordinates of brain position during MRI scanning, or baseline assessment centre. The e4 allele present vs. absent association was generally similar when we additionally corrected the WM hyperintensity volumes for total brain volume (total raw GM plus total raw WM): fully-adjusted standardised beta = 0.083, 95% CIs = 0.032 to 0.134, *P* = 0.001.

**Table 2:** Associations between *APOE* e4 presence and brain MRI variables (uncorrected for type-1 error)

	Partially adjusted				Fully adjusted			
	*b*	*Lower 95% CI*	*Upper 95% CI*	*p*	*b*	*Lower 95% CI*	*Upper 95% CI*	*p*
Association with *APOE* e4 presence								
gFA	−0.034	−0.092	0.024	0.255	−0.034	−0.092	0.025	0.259
gMD	0.027	−0.030	0.085	0.352	0.026	−0.031	0.084	0.369
Left hippocampus	−0.005	−0.058	0.049	0.861	−0.003	−0.057	0.051	0.911
Right hippocampus	−0.005	−0.058	0.048	0.858	0.002	−0.052	0.055	0.954
Total GM (normalised)	0.012	−0.031	0.055	0.585	0.010	−0.033	0.053	0.645
Total WM (normalised)	0.029	−0.025	0.082	0.294	0.019	−0.035	0.072	0.496
Total WM hyperintensities	**0.093**	**0.041**	**0.145**	**4 × 10**^**−4**^	**0.088**	**0.036**	**0.139**	**0.001**
*APOE* e4*age interaction								
gFA	<0.001	−0.008	0.008	0.985	<0.001	−0.008	0.009	0.924
gMD	0.002	−0.006	0.010	0.661	0.001	−0.007	0.009	0.768
Left hippocampus	−0.004	−0.012	0.003	0.252	−0.004	−0.012	0.004	0.291
Right hippocampus	−0.004	−0.012	0.004	0.308	−0.004	−0.011	0.004	0.342
Total GM (normalised)	0.001	−0.005	0.007	0.842	0.001	−0.005	0.007	0.638
Total WM (normalised)	−0.004	−0.011	0.004	0.340	−0.004	−0.012	0.003	0.258
Total WM hyperintensities	−0.001	−0.008	0.006	0.816	−0.002	−0.009	0.005	0.614

Partially adjusted: *APOE* e4 presence vs. absence plus 8 genetic principal components, assessment centre, genetic array, age in years and sex. Bold typeface denotes p < 0.05. Fully adjusted: (also) Townsend deprivation scores, self-reported diagnosed diabetes; hypertension; coronary heart disease, ever vs. never smoking.

*GM* grey matter; *WM* white matter; *FA* fractional anisotropy; *MD* mean diffusivity; *b* standardised beta; *CI* confidence interval; *APOE* apolipoprotein e; *MRI* magnetic resonance imaging

Note that Table 1 shows a significant difference between e4 absent vs. present in terms of total GM (*P* = 0.002), although this did not survive correction for relevant confounders (e.g. age and sex; see Table 2).

As additional exploratory analyses, we analysed the 22 individual tract-specific FA/MD values identified as most sensitive to age (Cox et al. 2016). There was one specific significant association, such that e4 carriers tended to have worse FA of the tract forceps major (fully adjusted standardised beta = −0.078, 95% CI = −0.138 to −0.018, *P* = 0.011). However, there were no e4 associations with MD, nor any interactions between e4 and age/age^2^ on FA/MD (*P* value range otherwise = 0.108 to 0.994; data available upon request). We re-ran the nominally significant e4 present vs. absent association with WM hyperintensity volumes, as an e4/e4 vs. non-e4 model: this was statistically significant (fully-adjusted model standardised beta = 0.217, 95% CI = 0.066 to 0.368, *P* = 0.005), with the caveat that there were relatively few e4 homozygotes (*n* = 189). The e4 allele present vs. absent association with WM hyperintensity volumes remained significant when corrected for type 1 error with FDR (fully adjusted q-value = 0.038) but the association with tract forceps major FA did not (fully adjusted q-value >0.05).

Supplementary Table 3 shows that compared with the full UK Biobank sample (after appropriate quality controlling as described in the methods), the imaging sample here had a similar frequency of *APOE* e4 but was slightly less deprived (imaging Townsend mean = −2.02, SD = 2.58 vs. -1.58, SD = 2.92, *P* < 0.001). Table 1 shows no significant effect of e4 presence vs. absence on log WM hyperintensity volumes, although this was significant in adjusted models. The main reason for this was the statistical adjustment for age at assessment (age at assessment vs. log hyperintensity volume standardised beta in multivariate fully adjusted model = 0.06, 95% CI = 0.06 to 0.07, P < 0.001), i.e. that when age was accounted for, there was a significant e4 effect. Full final model statistics are shown in Supplementary Table 4 and indicate that the e4/hyperintensity volume association was also independent of significant deleterious effects of male sex, diabetes, hypertension and smoking history.

## Discussion

This report examined the potential association between *APOE* e4 genotype and brain imaging phenotypes of relevance to cognitive aging and dementia. We have previously reported on the baseline cognitive data from UK Biobank (*n* = 111,739) and reported generally no *APOE* e4*age interaction (Lyall et al. 2016b), although the sensitivity of the tests used is unclear because they were novel, very brief, and suffered a degree of floor effects (Lyall et al. 2016a). We expected the brain imaging phenotypes to be more sensitive to age-related differences conditioned on *APOE* genotype. In terms of addition to the literature, this study is the largest single-site study of *APOE* e4 genotype and brain imaging metrics which are associated with cognitive aging.

With respect to our hypotheses, there was an association between *APOE* e4 genotype and significantly increased WM hyperintensity volume, such that e4 carriers exhibited 0.09 SDs greater load than non-carriers, and e4/e4 homozygotes around 0.22 SDs; this reinforces findings in a smaller meta-analysis (e.g. *n* = 4024) (Schilling et al. 2013). One limitation of our data is that the exact mechanisms which lead to rarefication of white matter tissue, are unclear, and may be due to different causes in different people (Wardlaw et al. 2013). The lack of a significant interaction with age provided no evidence that this effect was stronger at older ages; i.e. it was not the case that age and e4 genotype were synergistic. We found no significant associations or age interactions for *APOE* e4 with gFA, gMD, total GM, WM, or hippocampal volumes. The cross-sectional age range in the current sample, where most participants were aged 50 to 70 years, may limit our ability to find a significant interaction with age.

We found no significant interaction between cross-sectional older age, *APOE* e4 genotype and worse WM tract integrity (indexed by gFA/gMD) in around 8000 middle to older-aged adults. This is an unusually large non-consortium, single-scanner imaging genetics study, which allowed relatively high statistical power to reliably detect small effects; however we did not find any significant *APOE* e4 genotype-by-age interactions.

During exploratory analyses, we did find one relatively novel *APOE* e4 association with a specific tract FA value – namely in the tract forceps major. It is interesting that this tract was not assessed in the previously largest *APOE* e4/MRI study, using the Lothian Birth Cohort 1936 (*n* = 650) (Lyall et al. 2014). This demonstrates the importance of assessing as many specific WM tracts as can be reliably identified in imaging studies of *APOE* e genotype, and may indicate some differential sensitivity of some tracts (Cox et al. 2016; Lyall et al. 2014). This association did attenuate when corrected for type-1 error and may therefore be spurious. It is however worth noting that brain MRI phenotypes are usually strongly correlated (Cox et al. 2016) and generally speaking this can potentially make adjustment for type-1 overly cautious in some circumstances; independent replication in different cohorts is advised (Pike 2011).

We generally found no significant e4 genotype-by-age interactions on total GM, total WM, left or right hippocampal volumes or total WM hyperintensity volume. This is in line with some other findings; Lyall et al. (2013) reported that of six relatively large (N range 198 to 949) studies which examined *APOE* e4 genotype and hippocampal volumes, only two were statistically significant at *P* < 0.05. Generally, there is no evidence of a main effect of *APOE* e4 on GM volume in non-demented adults (Cherbuin et al. 2008). In terms of WM hyperintensities, one meta-analysis reported no significant association (*n* = 7351) however most studies were of observer-rated WH hyperintensities e.g. Fazekas scores (Paternoster et al. 2009) while another did report an association with continuous WM hyperintensity volumes (*n* = 8917) (Schilling et al. 2013) (standardised beta = 0.047, 95% CI = 0.0006 to 0.094, *P* = 0.05). Our results are therefore mostly in line with a generally null association between *APOE* e4 and cross-sectional brain volumes in middle age (around 60 years), but not WM hyperintensity volumes and this may suggest that at least part of *APOE* e4’s contribution to worse cognitive ability is via a cerebrovascular-type pathway; although the findings may also to an extent be secondary to prodromal AD.

While our findings are based on principally non-demented middle to older aged individuals, they are supportive of a vascular route from *APOE* e4 genotype to worse cognitive abilities. The vascular hypothesis of AD suggests significant cardiometabolic contributions to early AD neuropathology, particularly amyloid beta (Janota et al. 2016). It is possible that prodromal AD has not been fully accounted for in the current sample, although very few participants self-reported dementia at assessment.

In terms of limitations, this study tested for a cross-sectional effect of increasing age on brain WM microstructure and structural morphology; a longitudinal within-participants design may be more informative because it would minimize cohort effects associated with being born in different time periods (Anstey et al. 2003). UK Biobank will ultimately conduct longitudinal scanning in ~10,000 participants (Miller et al. 2016). The participants in the current study were of generally good health; we excluded participants who self-reported neurodegenerative diseases or those likely to affect the brain. However these diagnoses are not validated medically and we therefore cannot be certain of their accuracy; a recent analysis of self-reported rheumatoid arthritis in UK Biobank showed that only around half of participants that reported the chronic illness were on relevant medication, which puts the validity of the diagnoses into question (Siebert et al. 2016). Our analyses looked at relatively non-specific brain imaging phenotypes, as compared with for example hippocampal-subfields. Future studies will expand the range of phenotypes, as well as including a larger sample N once more UK Biobank participants have been scanned and their data released.

There may be some selection bias in the current sample, where primarily healthier older participants are likely to respond positively to invitation and attend assessment. It is also possible that this occurs across the whole sample with regards socioeconomic status, whereby more middle-class, professional people are liable to participate; we attempted to correct for this with Townsend scores however these are proxies for deprivation rather than being based on individual-level data. Generally, the rate of participants with exclusionary diseases were similar to the full UK Biobank cohort of approximately 500,000 – suggesting little bias in that regard between participants that attended baseline assessment from 2006 to 2010, vs. MRI scanning more recently. The current sample was slightly less deprived than the full UK Biobank dataset of n approximately 500,000. There may also be a degree of survival bias where the *APOE* e4 by age interaction is underestimated because the older people with a more deleterious effect of e4 are more likely to be missing (Heffernan et al. 2016): we saw no significant difference in e4 frequency by age group (<50; 50–59; ≥60) although there was a small but significant overall continuous effect where the e4 carriers were on average around half-a-year younger.

This report examined the effects of *APOE* e4 genotype on specific known substrates of worse cognitive aging however it was not totally exhaustive. For example, we did not investigate some brain phenotypes that have been reported to underlie some cognitive deficits, such as cortical thickness (Karama et al. 2014) nor did we examine structural brain metrics totally systematically and agnostically, e.g. voxel-based morphometry. Future research will take this more hypothesis-free approach in investigating *APOE* e4’s links to brain structure and its relevance to cognitive abilities.

Our principal finding was that *APOE* e4 significantly associated with increased WM hyperintensity volumes. In future, UK Biobank will include biomarker and serum lipid data. These data may be used to inform part of the causal chain from *APOE* genotype (a lipid transporter gene) to cognitive/brain phenotypes. While this report includes MRI and genetic data on around 10,000 participants from UK Biobank, ultimately around 100,000 will have data on these by around 2023 (Miller et al. 2016); this will permit even greater statistical power in future with which to detect such effects with greater reliability still.

## Electronic supplementary material

ESM 1 (DOCX 23 kb)

## References

[CR1] Alfaro-Almagro, F., Jenkinson, M., Bangerter, N. K., Andersson, J. L. R., Griffanti, L., Douaud, G., et al. (2017). Image processing and quality control for the first 10,000 brain imaging datasets from UK biobank. *NeuroImage*. 10.1016/j.neuroimage.2017.10.034. 10.1016/j.neuroimage.2017.10.034 PMC5770339 29079522

[CR2] Alloza C, Cox SR, Duff B, Semple SI, Bastin ME, Whalley HC, Lawrie SM (2016). Information processing speed mediates the relationship between white matter and general intelligence in schizophrenia. Psychiatry Research: Neuroimaging.

[CR3] Anstey KJ, Hofer SM, Luszcz MA (2003). Cross-sectional and longitudinal patterns of dedifferentiation in late-life cognitive and sensory function: The effects of age, ability, attrition, and occasion of measurement. Journal of Experimental Psychology: General.

[CR4] Bycroft, C., Freeman, C., Petkova, D., Band, G., Elliott, L. T., Sharp, K., et al. (2017). Genome-wide genetic data on ~500,000 UK biobank participants. *BiorXiv, 166298*. 10.1101/166298.

[CR5] Cherbuin N, Anstey KJ, Sachdev PS, Maller JJ, Meslin C, Mack HA, Wen W, Easteal S (2008). Total and regional gray matter volume is not related to APOE*E4 status in a community sample of middle-aged individuals. The Journals of Gerontology. Series A, Biological Sciences and Medical Sciences.

[CR6] Cox SR, Ritchie SJ, Tucker-Drob EM, Liewald DC, Hagenaars SP, Davies G, Wardlaw JM, Gale CR, Bastin ME, Deary IJ (2016). Ageing and brain white matter structure in 3,513 UK biobank participants. Nature Communications.

[CR7] Cox SR, Ritchie SJ, Dickie DA, Pattie A, Royle NA, Corley J, Aribisala BS, Harris SE, Valdés Hernández M, Gow AJ, Muñoz Maniega S, Starr JM, Bastin ME, Wardlaw JM, Deary IJ (2017). Interaction of APOE e4 and poor glycemic control predicts white matter hyperintensity growth from 73 to 76. Neurobiology of Aging.

[CR8] Davies G, Armstrong N, Bis JC, Bressler J, Chouraki V, Giddaluru S, Hofer E, Ibrahim-Verbaas CA, Kirin M, Lahti J, van der Lee SJ, le Hellard S, Liu T, Marioni RE, Oldmeadow C, Postmus I, Smith AV, Smith JA, Thalamuthu A, Thomson R, Vitart V, Wang J, Yu L, Zgaga L, Zhao W, Boxall R, Harris SE, Hill WD, Liewald DC, Luciano M, Adams H, Ames D, Amin N, Amouyel P, Assareh AA, Au R, Becker JT, Beiser A, Berr C, Bertram L, Boerwinkle E, Buckley BM, Campbell H, Corley J, de Jager PL, Dufouil C, Eriksson JG, Espeseth T, Faul JD, Ford I, Scotland G, Gottesman RF, Griswold ME, Gudnason V, Harris TB, Heiss G, Hofman A, Holliday EG, Huffman J, Kardia SLR, Kochan N, Knopman DS, Kwok JB, Lambert JC, Lee T, Li G, Li SC, Loitfelder M, Lopez OL, Lundervold AJ, Lundqvist A, Mather KA, Mirza SS, Nyberg L, Oostra BA, Palotie A, Papenberg G, Pattie A, Petrovic K, Polasek O, Psaty BM, Redmond P, Reppermund S, Rotter JI, Schmidt H, Schuur M, Schofield PW, Scott RJ, Steen VM, Stott DJ, van Swieten JC, Taylor KD, Trollor J, Trompet S, Uitterlinden AG, Weinstein G, Widen E, Windham BG, Jukema JW, Wright AF, Wright MJ, Yang Q, Amieva H, Attia JR, Bennett DA, Brodaty H, de Craen AJM, Hayward C, Ikram MA, Lindenberger U, Nilsson LG, Porteous DJ, Räikkönen K, Reinvang I, Rudan I, Sachdev PS, Schmidt R, Schofield PR, Srikanth V, Starr JM, Turner ST, Weir DR, Wilson JF, van Duijn C, Launer L, Fitzpatrick AL, Seshadri S, Mosley TH, Deary IJ (2015). Genetic contributions to variation in general cognitive function: A meta-analysis of genome-wide association studies in the CHARGE consortium (N=53949). Molecular Psychiatry.

[CR9] Debette S, Markus HS (2010). The clinical importance of white matter hyperintensities on brain magnetic resonance imaging: Systematic review and meta-analysis. BMJ.

[CR10] Eisenberg DTA, Kuzawa CW, Hayes MG (2010). Worldwide allele frequencies of the human apolipoprotein E gene: Climate, local adaptations, and evolutionary history. American Journal of Physical Anthropology.

[CR11] Faul F, Erdfelder E, Lang A-G, Buchner A (2007). G*power 3: A flexible statistical power analysis program for the social, behavioral, and biomedical sciences. Behavior Research Methods.

[CR12] Ferencz B, Laukka EJ, Lövdén M, Kalpouzos G, Keller L, Graff C, Wahlund LO, Fratiglioni L, Bäckman L (2013). The influence of APOE and TOMM40 polymorphisms on hippocampal volume and episodic memory in old age. Frontiers in Human Neuroscience.

[CR13] Griffanti L, Zamboni G, Khan A, Li L, Bonifacio G, Sundaresan V, Schulz UG, Kuker W, Battaglini M, Rothwell PM, Jenkinson M (2016). BIANCA (brain intensity AbNormality classification algorithm): A new tool for automated segmentation of white matter hyperintensities. NeuroImage.

[CR14] Heffernan AL, Chidgey C, Peng P, Masters CL, Roberts BR (2016). The neurobiology and age-related prevalence of the ε4 allele of apolipoprotein E in Alzheimer’s disease cohorts. Journal of Molecular Neuroscience.

[CR15] Heise V, Filippini N, Ebmeier KP, Mackay CE (2011). The APOE ɛ4 allele modulates brain white matter integrity in healthy adults. Molecular Psychiatry.

[CR16] Holtzman DM, Herz J, Bu G (2012). Apolipoprotein E and apolipoprotein E receptors: Normal biology and roles in Alzheimer disease. Cold Spring Harbor Perspectives in Medicine.

[CR17] Jack CR, Petersen RC, Xu YC, O’Brien PC, Waring SC, Tangalos EG (1998). Hippocampal atrophy and apolipoprotein E genotype are independently associated with Alzheimer’s disease. Annals of Neurology.

[CR18] Janota C, Lemere CA, Brito MA (2016). Dissecting the contribution of vascular alterations and aging to Alzheimer’s disease. Molecular Neurobiology.

[CR19] Jones DK, Knösche TR, Turner R (2013). White matter integrity, fiber count, and other fallacies: The do’s and don’ts of diffusion MRI. NeuroImage.

[CR20] Karama S, Bastin ME, Murray C, Royle NA, Penke L, Muñoz Maniega S, Gow AJ, Corley J, Valdés Hernández MC, Lewis JD, Rousseau MÉ, Lepage C, Fonov V, Collins DL, Booth T, Rioux P, Sherif T, Adalat R, Starr JM, Evans AC, Wardlaw JM, Deary IJ (2014). Childhood cognitive ability accounts for associations between cognitive ability and brain cortical thickness in old age. Molecular Psychiatry.

[CR21] Killiany RJ, Hyman BT, Gomez-Isla T, Moss MB, Kikinis R, Jolesz F, Tanzi R, Jones K, Albert MS (2002). MRI measures of entorhinal cortex vs hippocampus in preclinical AD. Neurology.

[CR22] Lutz, M. W., Crenshaw, D. G., Saunders, A. M., & Roses, A. D. (2010). Genetic variation at a single locus and age of onset for Alzheimer’s disease. Alzheimer's & Dementia: The Journal of the Alzheimer's Association;, 6(2), 125–131. 10.1016/j.jalz.2010.01.011. 10.1016/j.jalz.2010.01.011 PMC2874876 20298972

[CR23] Lyall DM, Royle NA, Harris SE, Bastin ME, Maniega SM, Murray C, Lutz MW, Saunders AM, Roses AD, del Valdés Hernández MC, Starr JM, Porteous DJ, Wardlaw JM, Deary IJ (2013). Alzheimer’s disease susceptibility genes APOE and TOMM40, and hippocampal volumes in the Lothian birth cohort 1936. PLoS One.

[CR24] Lyall DM, Harris SE, Bastin ME, Munoz Maniega S, Murray C, Lutz MW (2014). Alzheimer’s disease susceptibility genes APOE and TOMM40, and brain white matter integrity in the Lothian birth cohort 1936. Neurobiology of Aging.

[CR25] Lyall DM, Muñoz Maniega S, Harris SE, Bastin ME, Murray C, Lutz MW, Saunders AM, Roses AD, del C. Valdés Hernández M, Royle NA, Starr JM, Porteous DJ, Deary IJ, Wardlaw JM (2015). APOE/TOMM 40 genetic loci, white matter hyperintensities, and cerebral microbleeds. International Journal of Stroke.

[CR26] Lyall, D. M., Cullen, B., Allerhand, M., Smith, D. J., Mackay, D., Evans, J., Anderson, J., Fawns-Ritchie, C., McIntosh, A. M., Deary, I. J., & Pell, J. P. (2016a). Cognitive test scores in UK biobank: Data reduction in 480,416 participants and longitudinal stability in 20,346 participants. *PLoS One, 11*(4). 10.1371/journal.pone.0154222. 10.1371/journal.pone.0154222 PMC4844168 27110937

[CR27] Lyall DM, Ward J, Ritchie SJ, Davies G, Cullen B, Celis C, Bailey MES, Anderson J, Evans J, Mckay DF, Mcintosh AM, Sattar N, Smith DJ, Deary IJ, Pell JP (2016). Alzheimer disease genetic risk factor APOE e4 and cognitive abilities in 111,739 UK biobank participants. Age and Ageing.

[CR28] Manning EN, Barnes J, Cash DM, Bartlett JWJ, Leung KKK, Ourselin S (2014). APOE ε4 is associated with disproportionate progressive hippocampal atrophy in AD. PLoS One.

[CR29] Marioni RE, Campbell A, Scotland G, Hayward C, Porteous DJ, Deary IJ (2015). Differential effects of the APOE e4 allele on different domains of cognitive ability across the life-course. European Journal of Human Genetics.

[CR30] Miller KL, Alfaro-Almagro F, Bangerter NK, Thomas DL, Yacoub E, Xu J, Bartsch AJ, Jbabdi S, Sotiropoulos SN, Andersson JLR, Griffanti L, Douaud G, Okell TW, Weale P, Dragonu I, Garratt S, Hudson S, Collins R, Jenkinson M, Matthews PM, Smith SM (2016). Multimodal population brain imaging in the UK biobank prospective epidemiological study. Nature Neuroscience.

[CR31] Paternoster L, Chen W, Sudlow CLMM (2009). Genetic determinants of white matter hyperintensities on brain scans: A systematic assessment of 19 candidate gene polymorphisms in 46 studies in 19,000 subjects. Stroke; a Journal of Cerebral Circulation.

[CR32] Penke L, Maniega SM, Bastin ME, Valdés Hernández MC, Murray C, Royle NA, Starr JM, Wardlaw JM, Deary IJ (2012). Brain white matter tract integrity as a neural foundation for general intelligence. Molecular Psychiatry.

[CR33] Pievani M, Rasser PE, Galluzzi S, Benussi L, Ghidoni R, Sabattoli F, Bonetti M, Binetti G, Thompson PM, Frisoni GB (2009). Mapping the effect of APOE epsilon4 on gray matter loss in Alzheimer’s disease in vivo. NeuroImage.

[CR34] Pike N (2011). Using false discovery rates for multiple comparisons in ecology and evolution. Methods in Ecology and Evolution.

[CR35] Purcell S, Neale B, Todd-Brown K, Thomas L, Ferreira MAR, Bender D, Maller J, Sklar P, de Bakker PIW, Daly MJ, Sham PC (2007). PLINK: A tool set for whole-genome association and population-based linkage analyses. American Journal of Human Genetics.

[CR36] Reilly JF, Games D, Rydel RE, Freedman S, Schenk D, Young WG, Morrison JH, Bloom FE (2003). Amyloid deposition in the hippocampus and entorhinal cortex: Quantitative analysis of a transgenic mouse model. Proceedings of the National Academy of Sciences of the United States of America.

[CR37] Royle NA, Booth T, Valdés Hernández MC, Penke L, Murray C, Gow AJ, Maniega SM, Starr J, Bastin ME, Deary IJ, Wardlaw JM (2013). Estimated maximal and current brain volume predict cognitive ability in old age. Neurobiology of Aging.

[CR38] Scarmeas N, Stern Y (2006). Imaging studies and APOE genotype in persons at risk for Alzheimer’s disease. Current Psychiatry Reports.

[CR39] Schiepers OJG, Harris SE, Gow AJ, Pattie A, Brett CE, Starr JM, Deary IJ (2011). APOE E4 status predicts age-related cognitive decline in the ninth decade: Longitudinal follow-up of the Lothian birth cohort 1921. Molecular Psychiatry.

[CR40] Schilling S, DeStefano AL, Sachdev PS, Choi SH, Mather KA, DeCarli CD (2013). APOE genotype and MRI markers of cerebrovascular disease. *Neurology*.

[CR41] Schuff N, Woerner N, Boreta L, Kornfield T, Shaw LM, Trojanowski JQ, Thompson PM, Jack CR, Weiner MW, the Alzheimer's; Disease Neuroimaging Initiative (2009). MRI of hippocampal volume loss in early Alzheimer’s disease in relation to ApoE genotype and biomarkers. Brain : a Journal of Neurology.

[CR42] Siebert S, Lyall DM, Mackay DF, Porter D, McInnes IB, Sattar N, Pell JP (2016). Characteristics of rheumatoid arthritis and its association with major comorbid conditions: Cross-sectional study of 502 649 UK biobank participants. RMD Open.

[CR43] Slattery CF, Zhang J, Paterson RW, Foulkes AJM, Carton A, Macpherson K, Mancini L, Thomas DL, Modat M, Toussaint N, Cash DM, Thornton JS, Henley SMD, Crutch SJ, Alexander DC, Ourselin S, Fox NC, Zhang H, Schott JM (2017). ApoE influences regional white-matter axonal density loss in Alzheimer’s disease. Neurobiology of Aging.

[CR44] Sudlow C, Gallacher J, Allen N, Beral V, Burton P, Danesh J, Downey P, Elliott P, Green J, Landray M, Liu B, Matthews P, Ong G, Pell J, Silman A, Young A, Sprosen T, Peakman T, Collins R (2015). UK biobank: An open access resource for identifying the causes of a wide range of complex diseases of middle and old age. PLoS Medicine.

[CR45] Telford EJ, Cox SR, Fletcher-Watson S, Anblagan D, Sparrow S, Pataky R, Quigley A, Semple SI, Bastin ME, Boardman JP (2017). A latent measure explains substantial variance in white matter microstructure across the newborn human brain. Brain Structure & Function.

[CR46] Townsend P (1998). Townsend deprivation index.

[CR47] Wardlaw JM, Smith C, Dichgans M (2013). Mechanisms of sporadic cerebral small vessel disease: Insights from neuroimaging. The Lancet Neurology..

[CR48] Wisdom NM, Callahan JL, Hawkins KA (2011). The effects of apolipoprotein E on non-impaired cognitive functioning: A meta-analysis. Neurobiology of Aging.

[CR49] Zhang Y, Brady M, Smith S (2001). Segmentation of brain MR images through a hidden Markov random field model and the expectation-maximization algorithm. IEEE Transactions on Medical Imaging.

