# Supplementary material for: Association between APOE e4 and white matter hyperintensity volume, but not total brain volume or white matter integrity
Source: Brain Imaging Behav. 2019 Mar 22;14(5):1468–76. doi: 10.1007/s11682-019-00069-9 (PMC7572345; doi:10.1007/s11682-019-00069-9)
Supplement: Supplementary file 1 — (DOCX 23 kb) [file 11682_2019_69_MOESM1_ESM.docx]

**Supplementary Table 1** Excluded (self-reported) diseases.

Brain cancer/primary malignant tumour

Brain haemorrhage

Brain/intracranial abscess

Cerebral aneurysm

Cerebral palsy

Chronic/degenerative neurological problem

Dementia/Alzheimer disease/cognitive impairment

Encephalitis

Epilepsy

Fracture skull/head

Head injury

Infection of nervous system

Ischaemic stroke

Meningeal cancer/malignant meningioma

Meningioma (benign)

Meningitis

Motor neurone disease

Multiple sclerosis

Neurological injury/trauma

Neuroma (benign)

Other demyelinating condition

Other neurological problem

Parkinson disease

Spina bifida

Stroke

Subarachnoid haemorrhage

Subdural haematoma

Transient ischaemic attack

|  | ***APOE* e4 absent** | ***APOE* e4 present** |
| --- | --- | --- |
|  |  |  |
| <50 years | 325 (5.4%) | 127 (5.9%) |
| 50 to 59 | 1,908 (31.6%) | 717 (33.4%) |
| ≥60 | 3,807 (63.0%) | 1,303 (60.7%) |

**Supplementary Table 2** Frequency of *APOE* e4 genotype presence by age group: under 50; 50 to 59; 60 and over**.**

Statistics are total Ns (percentages). Overall 3x2 *chi* P value = 0.148.

**Supplementary Table 3** Deprivation metrics and *APOE* e4 frequencies in imaging sample vs. main UK Biobank.

|  | **Imaging sample (n=8,395)** | **UK Biobank sample (n=317,575*)** | **P-value** |
| --- | --- | --- | --- |
|  |  |  |  |
| E4 allele presence  (N; %) | 2,147 (26.2) | 83,605 (27.0) | 0.111 |
|  |  |  |  |
| Townsend score (mean; SD) | -2.02 (2.6) | -1.58 (2.9) | <0.001 |

The full UK Biobank sample is n=502,628: n=317,575 is after identical treatment as described in the methods, namely participants with *APOE* e genotypic data excluding those with non-white British ancestry, self-report vs. genetic sex mismatch, putative sex chromosomal aneuploidy, excess heterozygosity, and missingness rate >0.1, e2/e4 genotype and self-report neurological condition. A higher Townsend score means more deprivation.

**Supplementary Table 4** Fully-adjusted multivariate model statistics for association with log white matter hyperintensity volumes.

|  |  | **Confidence intervals** | |  |
| --- | --- | --- | --- | --- |
| **Model variable** | **Standardised beta** | **Lower** | **Upper** | **P-value** |
| *APOE* e4 (presence vs. absence) | 0.09 | 0.04 | 0.14 | 0.001 |
| PCA1 | <0.01 | -0.02 | 0.01 | 0.894 |
| PCA2 | -0.01 | -0.02 | 0.01 | 0.331 |
| PCA3 | 0.00 | -0.01 | 0.02 | 0.551 |
| PCA4 | 0.01 | -0.01 | 0.02 | 0.323 |
| PCA5 | <0.01 | <0.01 | 0.01 | 0.256 |
| PCA6 | -0.01 | -0.03 | <0.01 | 0.037 |
| PCA7 | 0.00 | -0.01 | 0.01 | 0.880 |
| PCA8 | <0.01 | -0.01 | 0.01 | 0.684 |
| Genotypic array (BiLeve vs. Axiom) | -0.03 | -0.10 | 0.05 | 0.512 |
| Age at MRI (years) | 0.06 | 0.06 | 0.07 | <0.001 |
| Sex (male vs. female) | 0.07 | 0.02 | 0.11 | 0.003 |
| Townsend | 0.01 | <0.01 | 0.01 | 0.196 |
| Diabetes (yes vs. no) | 0.20 | 0.09 | 0.31 | <0.001 |
| Hypertension | 0.28 | 0.23 | 0.34 | <0.001 |
| Coronary heart disease | 0.04 | -0.08 | 0.17 | 0.491 |
| Smoking history (ever vs. never) | 0.08 | 0.04 | 0.13 | <0.001 |
| Constant | -4.12 | -4.39 | -3.85 | <0.001 |

Betas reflect differences in log white matter hyperintensity volume, transformed to a per-SD scale.
